# Parental Attitudes and Tooth Brushing Habits in Preschool Children in Mangalore, Karnataka: A Cross-sectional Study

**DOI:** 10.5005/jp-journals-10005-1210

**Published:** 2013-10-14

**Authors:** Fawaz Pullishery, Ganesh Shenoy Panchmal, Rekha Shenoy

**Affiliations:** Postgraduate Student, Department of Public Health Dentistry Yenepoya Dental College, Mangalore, Karnataka, India, e-mail: drfawazp@gmail.com; Professor and Head, Department of Public Health Dentistry, Yenepoya Dental College, Mangalore, Karnataka, India; Reader, Department of Public Health Dentistry, Yenepoya Dental College, Mangalore, Karnataka, India

**Keywords:** Tooth brushing, Preschool children, Habits

## Abstract

**Background:** Adoption of consistent behavioral habits in childhood takes place at home, with the parents especially the mother, being the primary model for behavior. Tooth brushing habits which is learnt during early years of life, is deeply ingrained in the child's mind and it is expected that this leads to an adaptation of good oral hygiene in their later life.

**Objectives:** To assess the tooth brushing habits of preschool children and to determine the role and amount of supervision given to them by parents.

**Study design:** A pretested self-designed questionnaire was used to collect information from parents of 130 preschool children in Anganwadi and Kindergarten in Mangalore. Statistical analysis was done and Chi-square test was used.

**Results:** Tooth brushing habits in these children was started at a mean age of 22.4 months (SD 8.4).62% of the preschool children used toothbrush and toothpaste for cleaning teeth and brushing habits were mainly (84%) introduced by mothers. Seventy-one percent of the children were cooperative when they were introduced to tooth brushing.

**Conclusion:** Preschool children of Mangalore were introduced to tooth brushing at a mean age of 22.4 months. Mothers played a vital role in introducing and teaching the child how to brush. In children less than 10 months of age tooth brushing was not started at all.

**How to cite this article:** Pullishery F, Panchmal GS, Shenoy R. Parental Attitudes and Tooth Brushing Habits in Preschool Children in Mangalore, Karnataka: A Cross-sectional Study. Int J Clin Pediatr Dent 2013;6(3):156-160.

## INTRODUCTION

Tooth brushing habits which are learnt during early years of life, are deeply ingrained in the child's mind and this may leads to adoption of good oral hygiene methods in later life. Maintaining good oral health is important among preschool children as as prevalence of dental caries is found to be high in these children.^1,2^

Tooth brushing is a simple and effective way to remove plaque, thereby preventing dental caries and periodontal disease. There is little information in Mangalore on the practice of tooth brushing and when and how parents become aware that their child's mouth needs care and attention. Dentists can play an important role in the primary prevention of dental problems in young children through preventive treatments, risk assessment, and anticipatory guidance for parents regarding oral development, caries prevention and overall oral health.

Dental caries prevalence and severity in young children are high, and socioeconomic characteristics and dental utilization are important determinants of their dental caries experience.^3^ Maternal attitudes are likely to modify behaviors and thus play an important part in the uptake of favorable dental health practices. Studies indicating an increase in severity of dental caries also suggest mothers neither stress upon nor teach their children healthy lifestyles from birth.^4^ Young children's health behaviors and outcomes are influenced by their parent's knowledge and beliefs, which affect oral hygiene and healthy eating habits. Without basic knowledge of caries risk factors and how to take care of teeth, it is difficult to employ effective disease prevention strategies.^5^ The parents, especially mothers are influential figures in determining children's behavior. Mothers decide the kind of toothbrush, the amount of toothpaste used and the pattern of brushing their children adopt. Furthermore, the earlier the influence, the more likely it will determine the attitude and behavior of their children which may be difficult to change later in life.

It is indeed meaningful to analyze the factors which could influence the way in which preschool children learn tooth brushing. Hence, this study was undertaken to investigate:

1. How and when oral cleaning was introduced to preschool children and what were the methods used
2. To determine who was responsible for this introduction and supervision given to children
3. To compare the brushing frequency among preschool children.

## MATERIALS AND METHODS

The study population consisted of parents of 130 preschool children in four anganwadis and one kindergarten in Mangalore. A pretested questionnaire was used to collect sociodemographic details and information on oral hygiene practices. Parents were informed and a written consent was obtained prior to the study. Parents who gave consent for the study were included. Those who did not give consent were excluded from the study. Ethical clearance for the study was obtained from Yenepoya University Ethical Committee. A cross-sectional study was conducted among parents of 130 preschool children with a mean age of 28.40 months (SD 15). Both male and female children were examined but gender differences were not considered in this study. Preschool children those who were available at the time of the study were included for the study. A pretested questionnaire was used by calibrated examiner to record the information.

The data analysis included descriptive statistics. Associations between the tooth brushing with other variables were performed using a Chi-square test. The data was analyzed using SPSS version 16 (Chicago, IL, USA) and level of significance was set at p < 0.05.

## RESULTS

**Graph 1:**
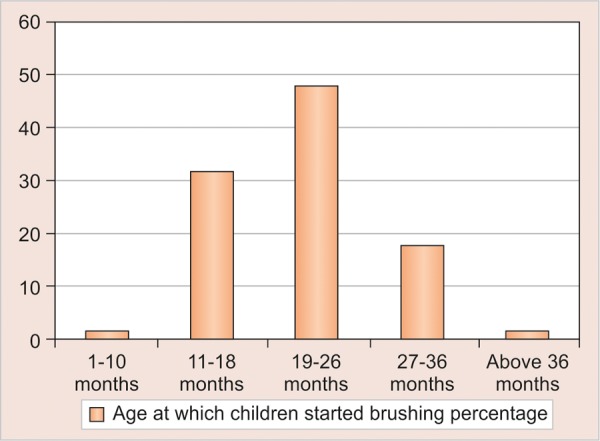
Age at which children started to brush their tooth

**Graph 2:**
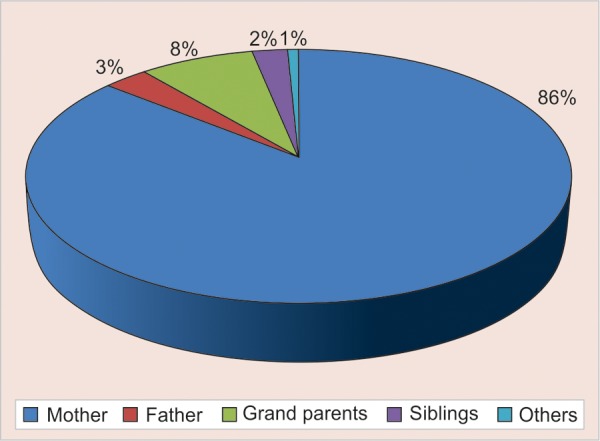
Person responsible for introducing tooth brushing in preschool children

**Graph 3:**
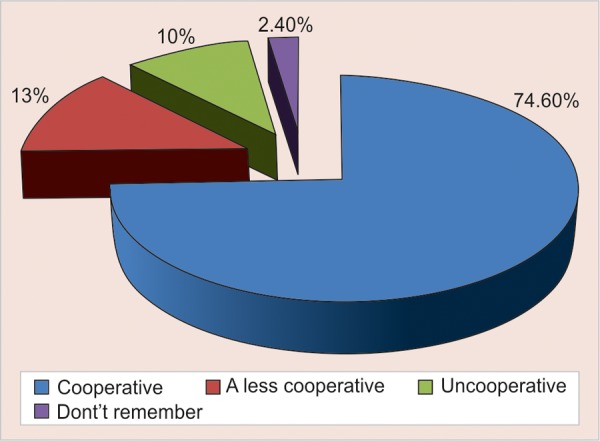
Behavior of child when brushing was introduced

The mean age of the preschool children was 28.40 (SD 15.3) months with median of 26 and ranged 8 to 68 month. The first tooth erupted in the oral cavity of these children was at a mean age (months) of 8.4 (SD 2.1) as reported by the parent through questionnaire. Preschool children started to brush their teeth at a mean age of 22.4 months (SD 8.4) (Table 1 and Graph 1) but used toothpaste only at a mean age of 27.3 (SD 7.6) months. The brushing habits were mainly introduced by mother (84%) than the father (4%) (Table 2 and Graph 2). The results showed that in terms of parents education, 31.53% of the mothers and 39.26% of the fathers had college education (Table 5 and Graph 5). It showed that 74.60% of children were cooperative when tooth brushing was introduced and only 10% were not cooperative (Table 3 and Graph 3). Younger mothers, mothers with higher level of education, urban residence and higher family income were seen to play a significant role in the use of brush and tooth paste by the child (p = 0.034).

Supervision for brushing was stopped at a mean age of 42 (SD 7.6) months. Positive association was observed between the age at which tooth brushing was started and the age of starting toothpaste in preschool children (p < 0.001). Twenty seven percent of preschool children of age between 13 and 72 months had received no instructions on tooth brushing. About 63% of preschool children 0 to 24 months of age were supervised.

**Graph 4:**
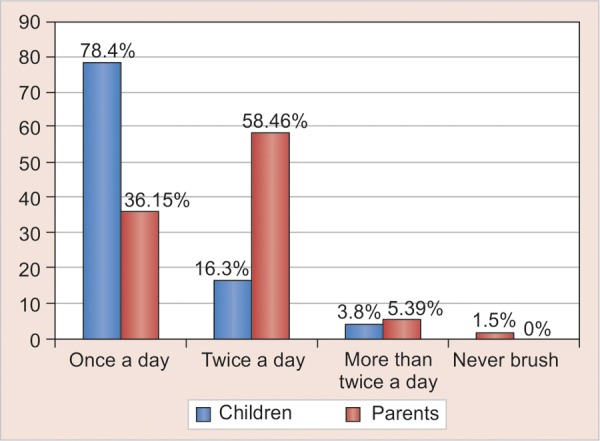
Frequency of brushing in children and parents

Tooth paste was used only for children >20 months ofage. Younger mothers, mothers with higher level of education, urban residence and higher family income were seen to play a significant role in the use of brush and toothpaste by the child (p = 0.034). The majority of parents (58.46%) brushed twice a day (78.4%), whereas in children, the majority brushed once a day, and quarter of the children were brushing when they remembered sometimes (Table 4 and Graph 4). The results of this study showed no statistically significant association between brushing frequencies of mothers and children (p = 0.591). On analysis of the item ‘who does the brushing’ in preschool children, the results suggested that in age groups 0 to 10 and 11 to 18, ‘mother’ was the most common response, which was followed by ‘father’ to be the second. Yet, for oldest age groups (19-26, 27-36 and >36), ‘child with the help of mothers’ were the most popular, which showed significant (p < 0.001) association with brushing supervision.

**Table 1:** **Table 1:** Age at which children started to brush their tooth

*Age (in months)*			*Total*
0-10 months	n	2	130
	%	1.53	
11-18 months	n	41	130
	%	31.53	
19-26 months	n	62	130
	%	47.69	
27-36 months	n	23	130
	%	17.69	
Above 36 months	n	2	130
	%	1.53	

**Table 2:** **Table 2:** Person responsible for introducing tooth brushing in preschool children

*Person responsible*	*n*	*%*
Mother	109	83.84
Father	4	3.07
Grand parents	10	7.69
Siblings	3	2.3
Others	1	0.76

**Table 3:** **Table 3:** Behavior of child when brushing was introduced

*Behavior of child*	*n*	*%*
Cooperative	97	74.60
Less cooperative	17	13
Un cooperative	13	10
Do not remember	3	2.4

## DISCUSSION

In this study, parents of 130 preschool children were interviewed with a mean age of 28.40 months. It was decided to recruit our sample from Anganwadis and Kindergartens in Mangalore, as it represented children in the age group that we were interested to study. Among the parents of the pre-school children, mothers were interviewed in most cases. Tooth brushing was started in preschool children at the age of 12 to 28 months (mean age being 22.4 months), whereas toothpaste was introduced later at a mean age of 19.7 months. This indicates that parents usually mothers started to brush their children's teeth much later than they had noticed the eruption of the first tooth in the oral cavity at a mean age of 8.4 (SD 2.1) months. Majority (more than 50%) of preschool children started to use toothpaste only at age of 24 months.

**Table 4:** **Table 4:** Frequency of tooth brushing in preschool children and parents

	*Children*			*Parents*	
*Frequency*	*n = 130*	*%*		*n = 130*	*%*
Once a day	102	78.4		47	36.15
Twice a day	21	16.3		76	58.46
More than twice a day	5	3.8		7	5.39
Never brush	2	1.5		0	0

In this study, introduction to tooth brushing in pre-school children was done mainly by the mothers. The results of this study support findings of Khadri et al^1^ which emphasized on the role of the mother in introducing the child to tooth brushing. It is interesting to note the way in which tooth brushing was introduced among these children were mainly by toothbrush (47%) and by traditional methods (3%). Comparing tooth brushing frequency of mothers to their preschool children, there is a lack of correlation between the brushing behaviors of mothers and their preschool children in this study. This is not in line with previous studies which indicated that parents' oral behavior had a direct influence on the number of decayed teeth in their children (Okada et al).^2^

Tooth brushing in preschool children was supervised by mothers and this percentage increased with the child's age, but a small proportion of children were not brushing their teeth at all in this study. In reference to brushing supervision, other studies have also reported similar findings of Hashim et al).^3^ Although the results of this study showed lack of significant correlation between brushing frequency of mothers to their preschool children, it should be noted that (Table 4) all of the parents brushed either once, twice or thrice a day. This good oral hygiene practice in their parents has resulted in 75% of preschool children to brush their teeth. The lack of dental health education to preschool children and their parents could be the reason for a big proportion of children brushing their teeth only once a day, infrequently or not at all.^4^

**Table 5:** **Table 5:** Level of education of parents

*Level of education*	*Father*			*Mother*	
	*n*	*%*		*n*	*%*
No education	1	0.76		2	1.53
Primary school	28	21.53		38	29.23
Secondary school	35	26.92		37	28.48
High school	51	39.26		41	31.53
College education	15	11.53		12	9.23

**Graph 5:**
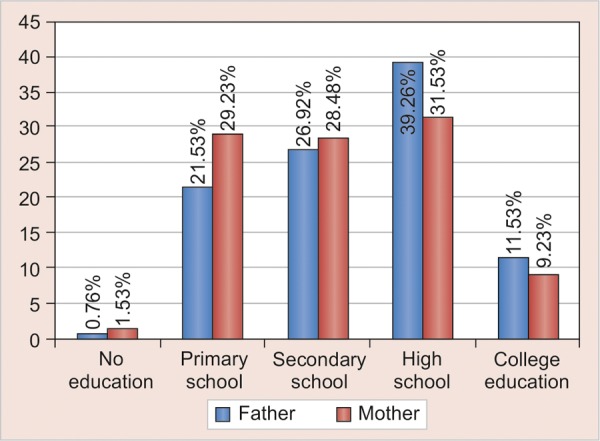
Level of education of parents

It is interesting to observe that tooth brushing which was exclusively carried out by the child were few; whereas in most cases, brushing was done with the help of mother. This finding is not in correlation with previous report where 40% of preschool children refused parents' help for brushing (Finlayson et al).^5^ Although, analyzing the reasons given by parents for not supervising tooth brushing were the child knew how to brush, large family size and, in few cases, child wanted to brush their teeth by themselves.^6^ Most of the mothers have a very little knowledge regarding the oral hygiene practices which includes brushing, flossing, use of fluoride, etc. These findings correlate with the findings of Suresh et al.^7^ There was statistically difference between observed tooth brushing habits and reported by parents especially mothers which is similar to the findings of Mohebbi et al^8^ Martins et al.^9^ In this study, mothers' preventive dental behavior was one of the factors which influenced the child's dental health and these findings are similar to the study conducted by Chen MS.^10^ Younger mothers and mothers with high level of education have a better knowledge on use of toothbrush, toothpaste and these findings correlate with the previous studies done by Sufia et al^11^ and Hosani et al.^12^

## CONCLUSION

In summary, preschool children of Mangalore, Karnataka were introduced to mouth cleaning by toothbrush at a mean age of 16 months. Mothers played a pivotal role in introducing and teaching the child how to brush. There was no positive correlation between the brushing behavior of the parents and among preschoolers. When preschooler was above 25 months of age tooth brushing was carried out by the child with the help of parents.

The results of this study are important as they indicate that future health education programs should reinforce existing behavior by supporting it with specific technical advice for brushing, including information on when, how long and how often to brush. Also the reasons for brushing the teeth and the age when brushing should begin could be more clearly defined. Importance of oral health education and promotion to parents by health personnel during routine health check-ups and dental visits of preschool children should be implemented and also importance of promoting earlier start of use of fluoride toothpaste to prevent dental caries should be recommended.

## References

[1] Khadri FA, Gopinath VK, Hector MP, Davenport ES (2010). How pre- school children learn to brush their teeth in Sharjah, United Arab Emirates.. Int J Paediatr Dent.

[2] Okada M, Kawamura M, Kaihara Y, Matsuzaki Y, Kuwahara S, Ishidori H, Miura K (2002). Influence of parents' oral health behaviour on oral health status of their school children: an exploratory study employing a causal modeling technique.. Int J Paediatr Dent.

[3] Hashim R, Thomson WM, Ayers KM, Lewsey JD, Awad M (2006). Dental caries experience and use of dental services among pre- school children in Ajman, UAE.. Int J Paediatr Dent.

[4] Huebner CE, Riedy CA (2010). Behavioral determinants of brushing young children's teeth: implications for anticipatory Guidance.. Pediatr Dent.

[5] Finlayson TL, Siefert K, Ismail AI, Sohn W (2007). Maternal self- efficacy and 1 to 5 year-old children's brushing habits.. Community Dent Oral Epidemiol.

[6] Zeedyk MS, Longbottom C, Pitts NB (2005). Toothbrushing practice of parents and toddlers: study of home-based videotaped session.. Caries Res.

[7] Suresh BS, Ravishankar TL, Chaitra TR, Mohapatra AK, Gupta V (2010). Mother's knowledge about pre-school child's oral health.. J Indian Soc Pedod Prev Dent.

[8] Mohebbi SZ, Virtanen JI, Murtomaa H, Vahid-Golpayegani M, Vehkalahti MM (2008). Mothers as facilitators of oral hygiene in early childhood.. Int J Paediatr Dent.

[9] Martins CC, Oliveira MJ, Pordeus IA, Paiva SM (2011). Comparison between observed children's tooth brushing habits and those reported by mothers.. BMC Oral Health.

[10] Chen MS (1986). Children's preventive dental behaviour in relation to their mothers' socioeconomic status, health beliefs and dental behaviour.. ASDC J Dent Child.

[11] Sufia S, Khan AA, Chaudhary S (2009). Maternal factors and child's dental health.. J Oral Health Comm Dent.

[12] Al-Hosani E, Rugg-Gunn A (1998). Combination of low parental educational attainment and high parental income related to high caries experience in preschool children in Abu Dhabi.. Community Dent Oral Epidemiol.

